# α-Actinin-3: Why Gene Loss Is an Evolutionary Gain

**DOI:** 10.1371/journal.pgen.1004908

**Published:** 2015-01-15

**Authors:** Niklas Ivarsson, Håkan Westerblad

**Affiliations:** Department of Physiology and Pharmacology, Karolinska Institutet, Stockholm, Sweden; Stanford University School of Medicine, United States of America

## Introduction

Large-scale sequencing of human populations has revealed many regions of the genome that have undergone positive selection during recent human evolution [1]. For most such regions, the genes and the nucleotide variants under selection are challenging to identify, and one can only guess about the cellular and physiological mechanisms. In this issue of *PLOS Genetics*, Head et al. [2] shed light on this question for one of the most fascinating examples of selection, in part because the variant undergoing selection is a loss-of-function, and in part because it was discovered long before the human genome sequence was completed.

Originally identified during a search for muscular dystrophy defects [3], deficiency of α-actinin-3 later turned out to be surprisingly common [4]. Roughly 18% of the world population is homozygous for a nonsense mutation (R577X) in ACTN3 deficiency, and the derivative allele (*ACTN3* 577xx) frequency correlates with greater latitude and lower temperature [5]. There is an intriguing correlation with athletic performance—the derivative allele is overrepresented among elite marathoners and other endurance athletes, but underrepresented among elite sprinters—indeed, the ancestral allele has been referred to as “the gene for speed” [6]. The evidence for positive selection of the derivative allele in European and East Asian populations is strong, but the phenotype being selected is uncertain and the underlying cell biology is even less clear. The article by Head et al. [2] provides some clarity and, together with earlier work from our group (Bruton et al. [7]), a unifying hypothesis.

## Background

To put the work on mechanism into context, it is helpful to review some of the basics of ACTN3 biology. The *ACTN3* gene is only expressed in glycolytic, fast-twitch (type II) skeletal muscle fibers, where it binds to actin and is part of the Z-line in the sarcomere structure [8]. Considerable insight into function has come from knockout mice: fast-twitch muscle fibers of *Actn3* knockout (KO) mice have increased aerobic capacity with increased citrate synthase (CS) activity and higher expression of mitochondrial proteins, such as cytochrome c oxidase and porin [4]. The *Actn*3 KO mice can cover more distance on a treadmill, and therefore exhibit adaptations also observed in response to endurance exercise [9].

One interesting aspect of *Actn3* KO muscle is an increase in calcineurin (CaN) signaling [10]. CaN, together with calmodulin kinase (CaMK), acts as a Ca^2+^ decoder that responds to increases in Ca^2+^ and trigger intracellular signaling [11]. Wright et al. showed that mitochondrial biogenesis is activated in skeletal muscle by artificially increasing cytosolic [Ca^2+^] with caffeine; e.g., increases in citrate synthase and cytochrome c oxidase mRNA were observed 24 hours after caffeine exposure [12]. They also observed an increase in peroxisome proliferator-activated receptor ɣ coactivator 1-α (PGC-1α) [12], which is regarded as key promoter of mitochondrial biogenesis [13, 14].

Work from our group (Bruton et al.) showed that in cold-exposed mice, there was also a link between sarcoplasmic reticulum (SR) Ca^2+^ leak and mitochondrial biogenesis. Non-shivering muscles of cold-exposed mice displayed increased expression of PGC-1α with subsequent increases in citrate synthase activity and endurance [7].

## Bringing It All Together

**Figure 1:**
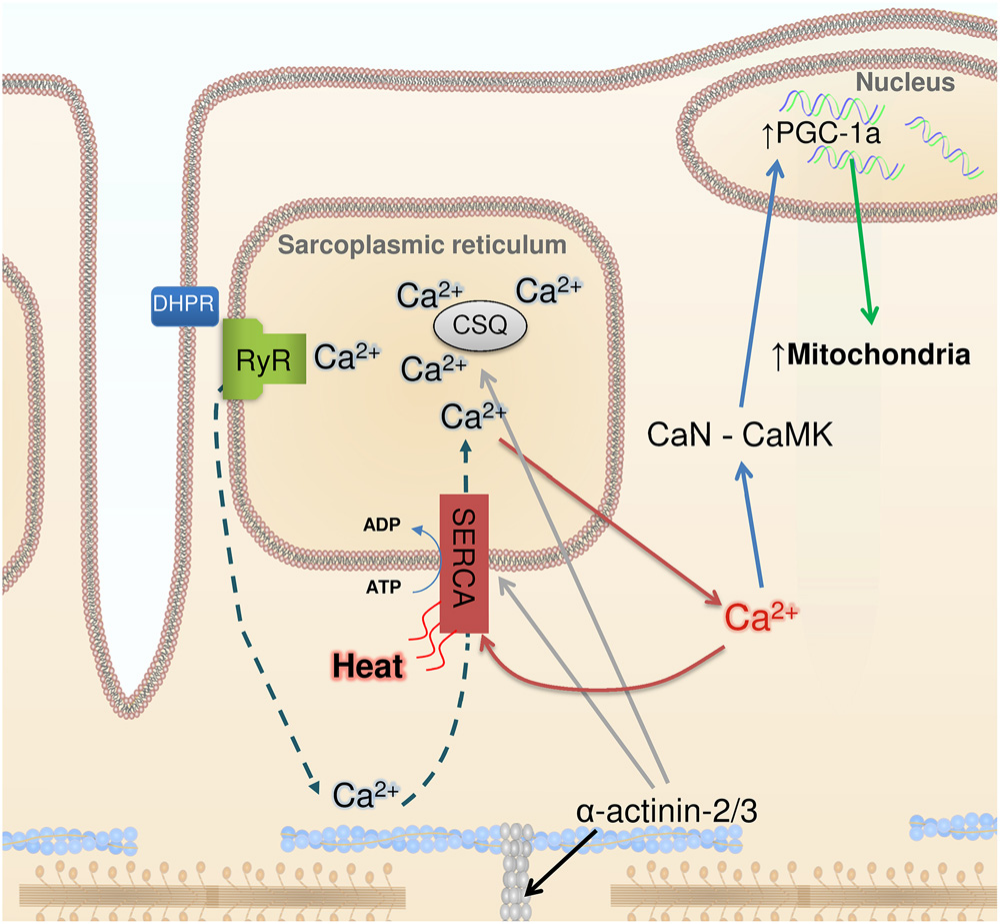
Ca^2+^, heat, and mitochondrial biogenesis. The contraction of skeletal muscle fibers is initiated by sarcoplasmic reticulum (SR) Ca^2+^ release via the ryanodine receptors (RyR), which is triggered by action potential activation of the transverse tubular voltage sensors, the dihydropyridine receptors (DHPR). Ca^2+^ activates the contractile machinery and is subsequently pumped back into the SR via SERCA (dashed arrows). α-Actinin 3 deficiency results in increased protein expression of SERCA and the SR Ca^2+^ buffers calsequestrin (CSQ) (grey arrows) and sarcalumenin (not shown). These changes are accompanied by increased SR Ca^2+^ leak and, subsequently, increased Ca^2+^ reuptake (red arrows), which generates heat. Increased [Ca^2+^] in the cytosol can trigger calcineurin (CaN) and calmodulin kinase (CaMK), resulting in PGC-1α activation (blue arrows) and subsequent mitochondrial biogenesis (green arrow).

In this issue of *PLOS Genetics*, Head et al. [2] observed marked changes in cellular Ca^2+^ handling in fast-twitch muscles of *Actn3* KO mice. These muscles expressed more of the SR Ca^2+^ ATPase 1 (SERCA1) and the SR Ca^2+^ buffering proteins calsequestrin 1 and sarcolumenin. Muscle fibers of *Actn3* KO mice showed 3- to 4-fold increases in SR Ca^2+^ leak and Ca^2+^ reuptake. Moreover, cytoplasmic Ca^2+^ transients were better maintained during repeated tetanic stimulation, which is in accordance with previously published data showing increased fatigue resistance in muscles of *Actn3* KO mice (Fig. 1).

Head et al. highlight the similar adaptations in *Actn3* KO muscles and non-shivering muscles of cold-acclimated mice, which also show increased SR Ca^2+^ leak and are more fatigue resistant [7]. An increased SR Ca^2+^ leak would require increased SR Ca^2+^ re-uptake and increased SERCA1 ATP hydrolysis, which would generate heat. Thus, in addition to heat from activation of brown adipose tissue [15], fatigue-resistant muscle fibers with leaky SR would contribute to non-shivering thermogenesis, providing a tentative explanation for the evolutionary advantage of carrying the *ACTN3* 577xx gene in a cold climate.

## Unanswered Questions and Future Perspectives

From a cell biologic perspective, the source of the SR Ca^2+^ leak in *Actn3* KO muscle is not yet clear. Head et al. [2] suggest that the major source is via SERCA [16]; alternatively, it might be due to destabilized SR Ca^2+^ release channel (ryanodine receptor, RyR) protein complexes [7, 17, 18]. Regardless, the SR Ca^2+^ leak seems to enhance the oxidative capacity of muscle in a number of settings: development, as with the *Actn3* KO mice; stress, such as cold exposure; and, possibly, endurance exercise.

From an evolutionary perspective, the SR Ca^2+^ leak may be good for ancestral humans in cold climates and good for endurance athletes, but it is also known to be deleterious in aging-associated muscle weakness [19], in muscular dystrophies [18], and in response to excessive endurance training (“overtraining”) [17]. In this respect, the evolutionary balance between the functional and non-functional *ACTN3* alleles may be “playing with fire”, as exemplified by results from cold-exposed mice. In these animals, we noted that minor modifications in the RyR protein complex were accompanied by larger cytosolic [Ca^2+^] during contractions and increased fatigue resistance [7] in non-shivering muscle. In more stressed, shivering muscle, however, severe RyR modifications led to decreased tetanic [Ca^2+^] and muscle weakness [20].

Human evolution and athletic performance are fascinating, but the findings of Head et al. provide additional avenues for future studies with important implications for human health, since the benefits of improved mitochondrial function span far beyond increased exercise capacity. Obesity and the metabolic syndrome are associated with impaired mitochondrial function, and of course, constitute a widespread and rapidly increasing health problem. Could strategies that phenocopy the effects of the *ACTN3 577xx* allele promote increased energy expenditure and improved mitochondrial function without requiring an increase in physical activity? Perhaps treatments to induce a controlled SR Ca^2+^ leak provide such an opportunity, but then the risk of causing impaired muscle function due to excessive Ca^2+^ leakage has to be overcome.
